# Supplementary material for: Facilitating alkaline hydrogen evolution reaction on the hetero-interfaced Ru/RuO2 through Pt single atoms doping
Source: Nat Commun. 2024 Feb 16;15:1447. doi: 10.1038/s41467-024-45654-9 (PMC10873302; doi:10.1038/s41467-024-45654-9)
Supplement: Supplementary file 1 — Supplementary Information [file 41467_2024_45654_MOESM1_ESM.pdf]

Supplementary Information for

Facilitating alkaline hydrogen evolution reaction on the hetero-interfaced Ru/RuO_2 through Pt single atoms doping

Zhu *et al.*

Supplementary Figures

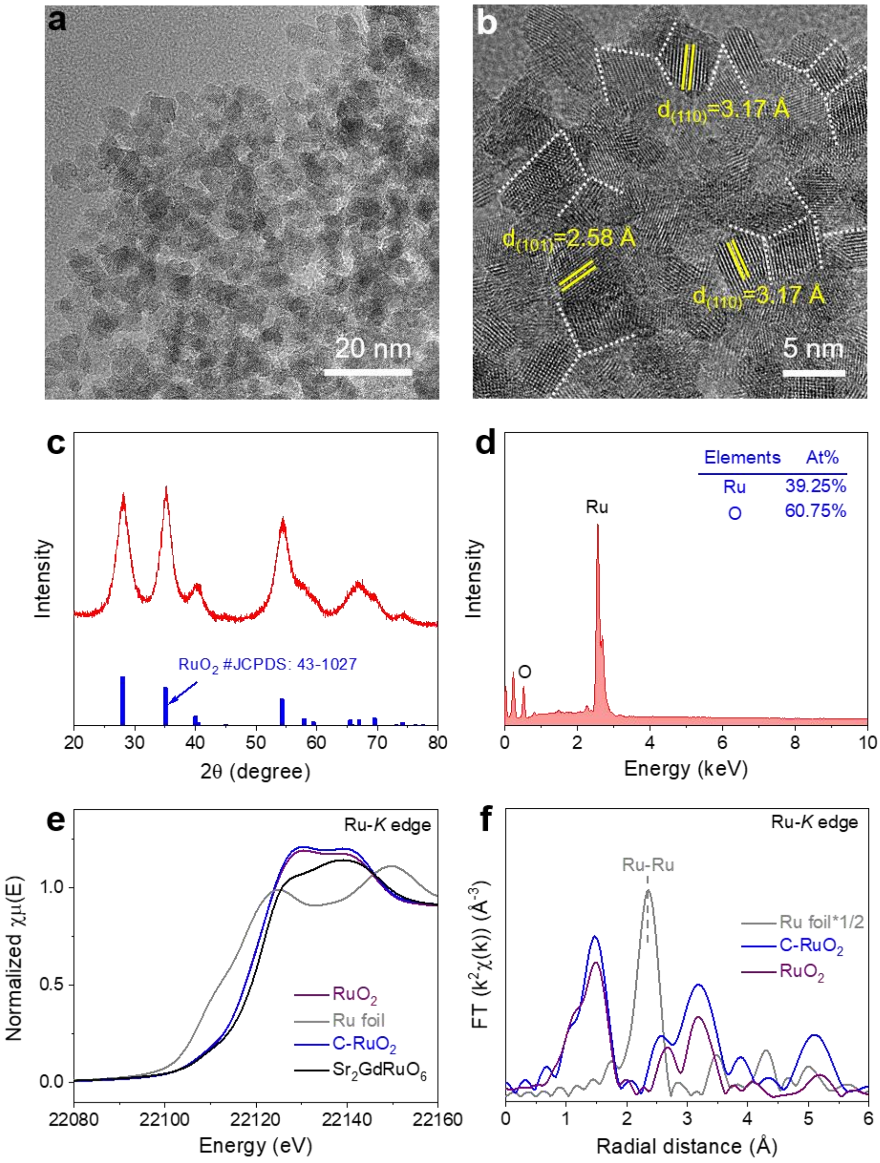

Supplementary Figure 1. **Characterizations of synthesized RuO_2.** (a) TEM, (b) HR-TEM images, (c) XRD pattern and (d) SEM-EDS spectrum of synthesized RuO_2. (e) The normalized Ru *K*-edge XANES spectra of RuO_2, Ru foil, commercial RuO_2 (C-RuO_2) and Sr_2GdRuO_6. (f) Ru *K*-edge EXAFS spectra of RuO_2, Ru foil and C-RuO_2. White dash lines represent the grain boundaries in RuO_2.

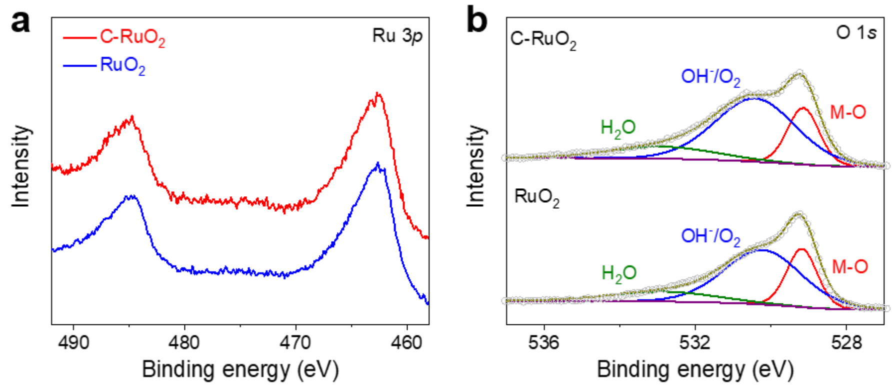

Supplementary Figure 2. **XPS spectra of synthesized RuO_2 and C-RuO_2.** (a) Ru 3*p* and (b) O 1*s* XPS spectra of the C-RuO_2 and synthesized RuO_2.

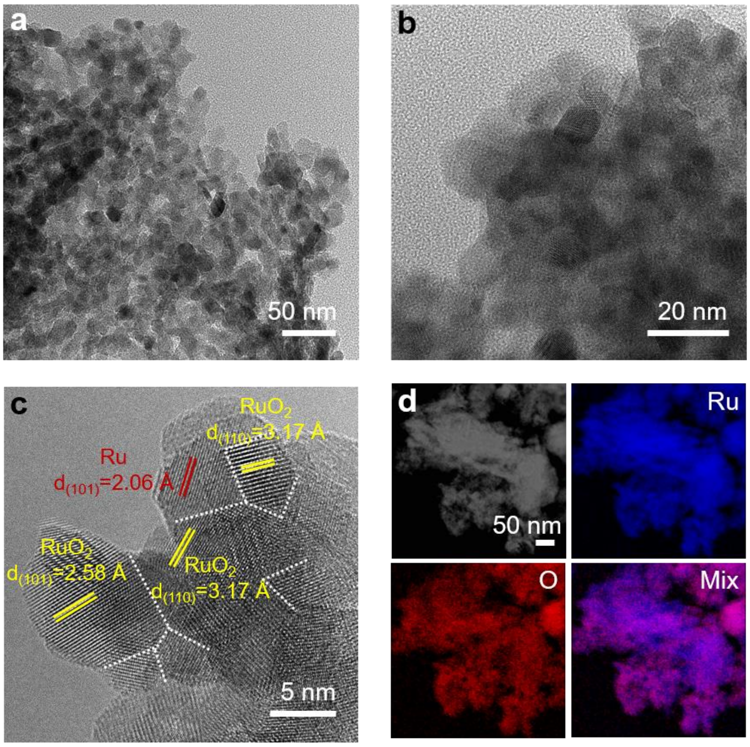

Supplementary Figure 3. **Electron microscope characterizations of Ru/RuO_2.** (a, b) TEM, (c) HR-TEM images, and (d) elemental mappings of Ru/RuO_2. White dash lines represent the grain boundaries in Ru/RuO_2.

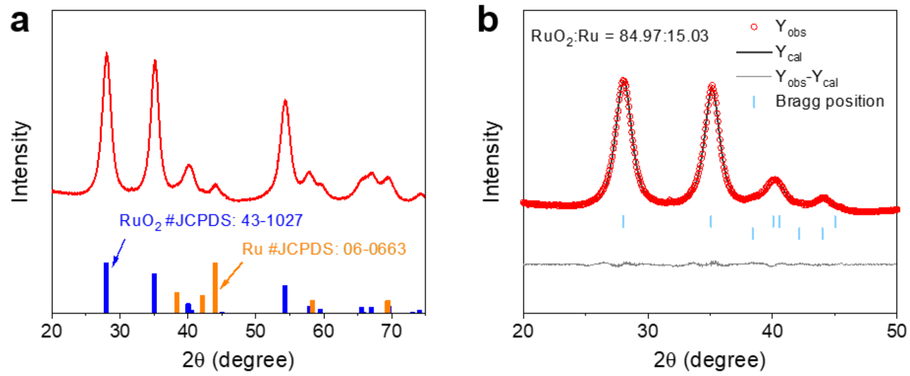

Supplementary Figure 4. **XRD patterns of Ru/RuO$_2$.** (a) XRD and (b) Rietveld refinement patterns of Ru/RuO$_2$.

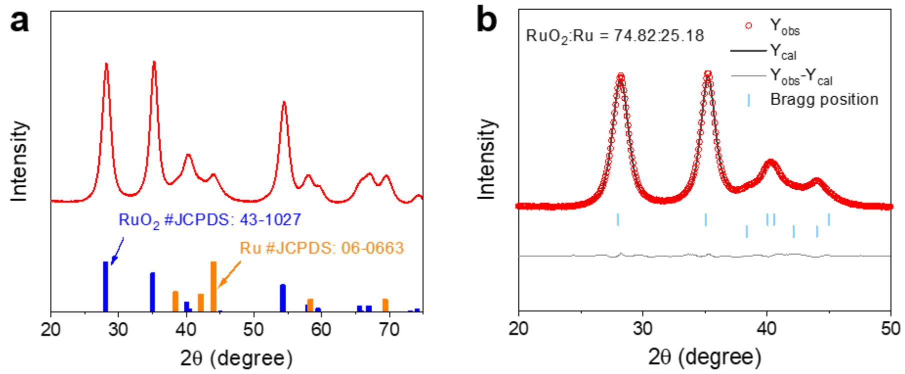

Supplementary Figure 5. **XRD patterns of Pt-Ru/RuO$_2$.** (a) XRD and (b) Rietveld refinement patterns of Pt-Ru/RuO$_2$.

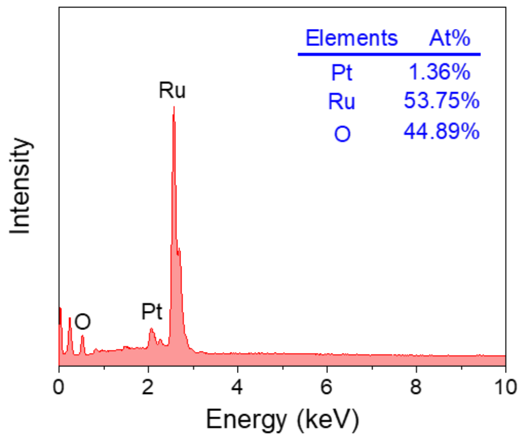

Supplementary Figure 6. **SEM-EDS pattern of Pt-Ru/RuO_2.**

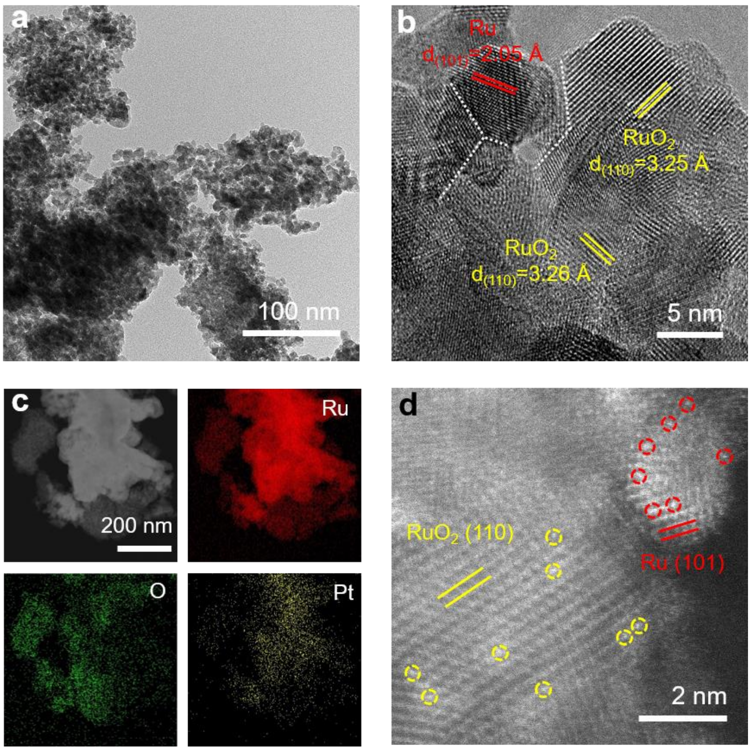

Supplementary Figure 7. **Electron microscope characterizations of Pt-Ru/RuO_2.** (a) TEM, (b) HR-TEM images, (c) elemental mappings and (d) AC HAADF-STEM image of Pt-Ru/RuO_2. White dash lines represent the grain boundaries in Pt-Ru/RuO_2.

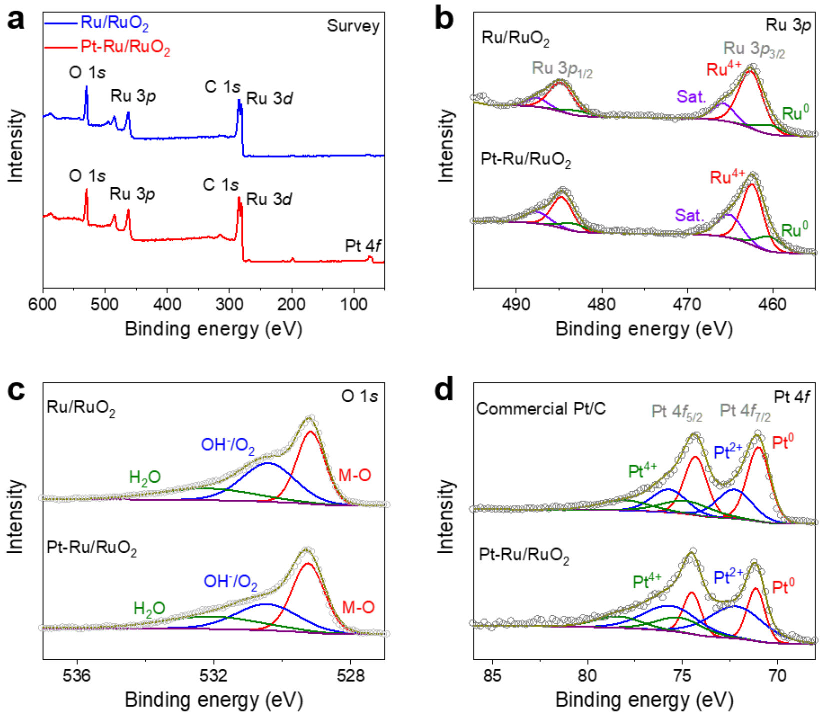

Supplementary Figure 8. **XPS spectra of Pt-Ru/RuO_2 and references.** The (a) survey, (b) Ru 3*p*, and (c) O 1*s* XPS spectra of Ru/RuO_2 and Pt-Ru/RuO_2. (d) Pt 4*f* XPS spectra of commercial Pt/C and Pt-Ru/RuO_2.

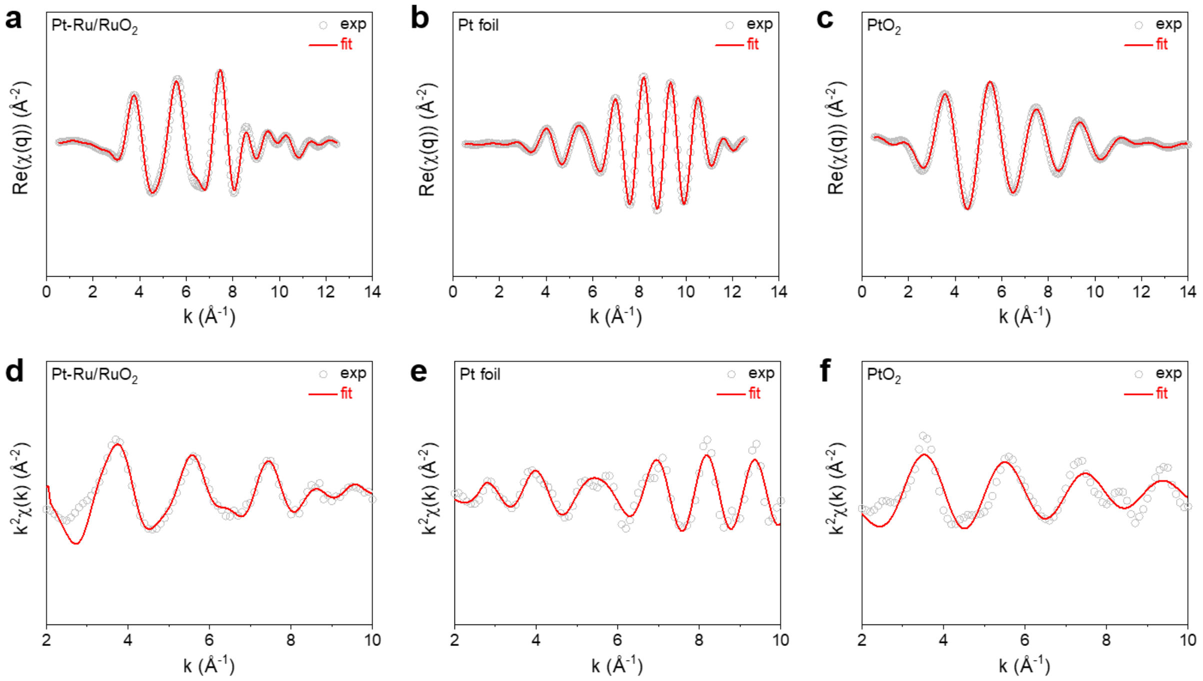

Supplementary Figure 9. **Pt L_3-edge EXAFS fitting spectra of Pt-Ru/RuO_2 and references.** (a-c) k^2-weighted q-space Pt L_3-edge experimental and fitting spectra of Pt-Ru/RuO_2 and references. (d-f) k^2-weighted k-space Pt L_3-edge experimental and fitting spectra of Pt-Ru/RuO_2 and references.

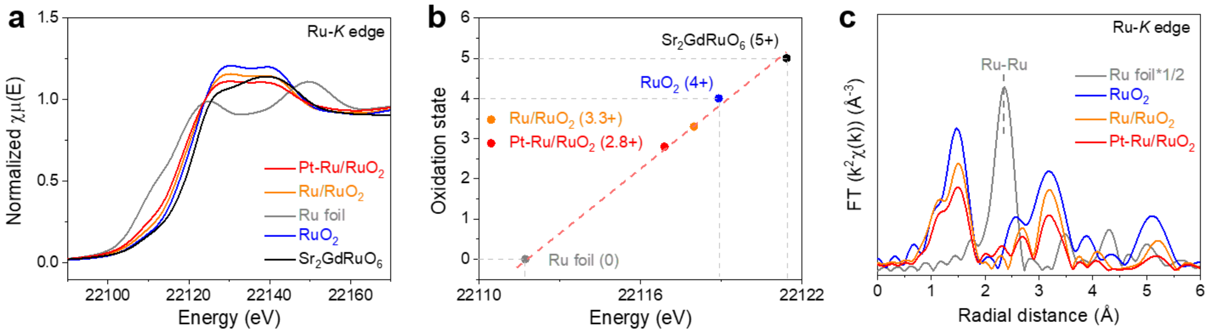

Supplementary Figure 10. Ru *K*-edge XAS spectra of Pt-Ru/RuO_2 and references. (a) The normalized Ru *K*-edge XANES spectra of Pt-Ru/RuO_2, Ru/RuO_2, Ru foil, RuO_2 and Sr_2GdRuO_6. (b) Ru valence state-absorption energy standard curves determined by Ru foil (Ru^0), RuO_2 (Ru^{4+}) and Sr_2GdRuO_6 (Ru^{5+}), and the calculated Ru valence states on Pt-Ru/RuO_2 and Ru/RuO_2. (c) The normalized Ru *K*-edge EXAFS spectra of Pt-Ru/RuO_2, Ru/RuO_2, Ru foil and RuO_2.

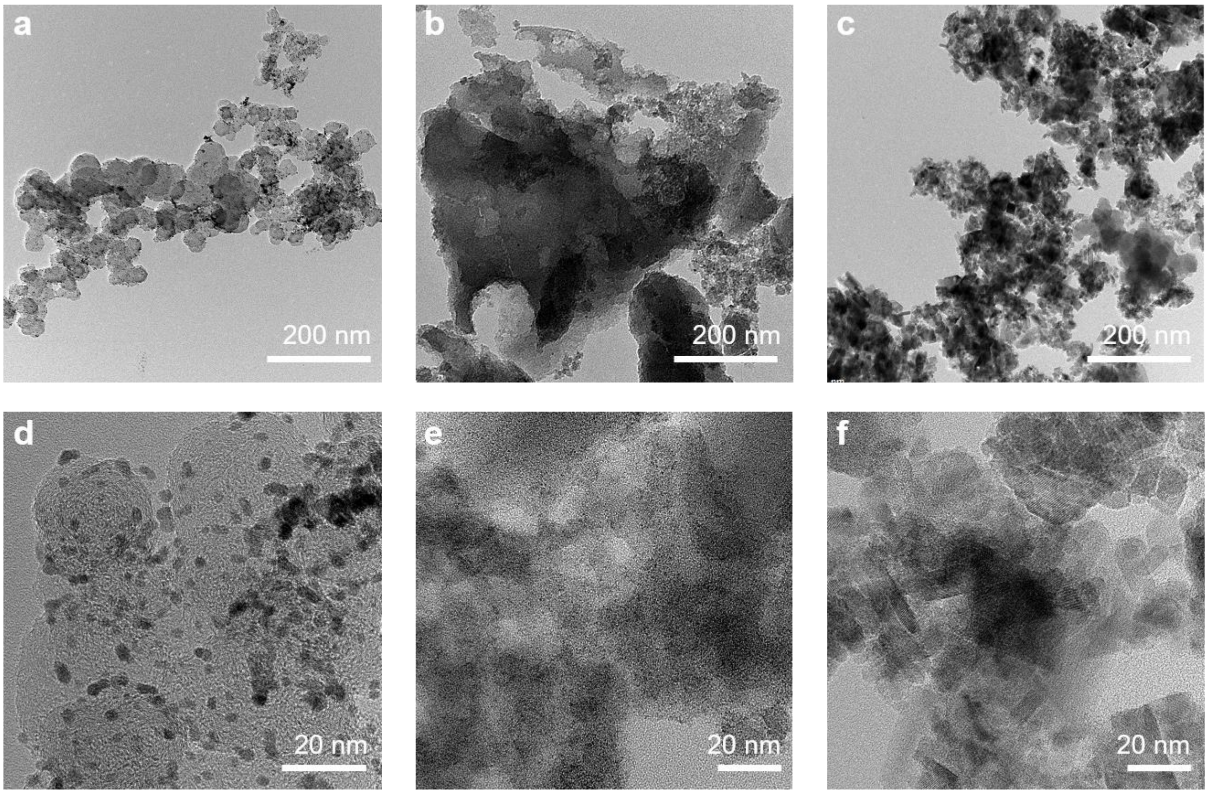

Supplementary Figure 11. TEM images of commercial references. The TEM images of (a, d) commercial Pt/C, (b, e) Ru/C, and (c, f) C-RuO_2 at the scales of 200 nm and 20 nm, respectively.

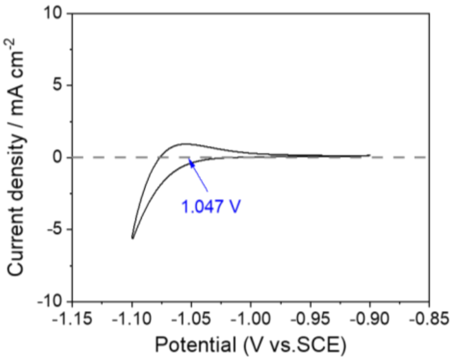

Supplementary Figure 12. Potential calibration of the reference electrode. Potential calibration of the calomel reference electrode at room temperature in H_2-saturated 1 M KOH solution.

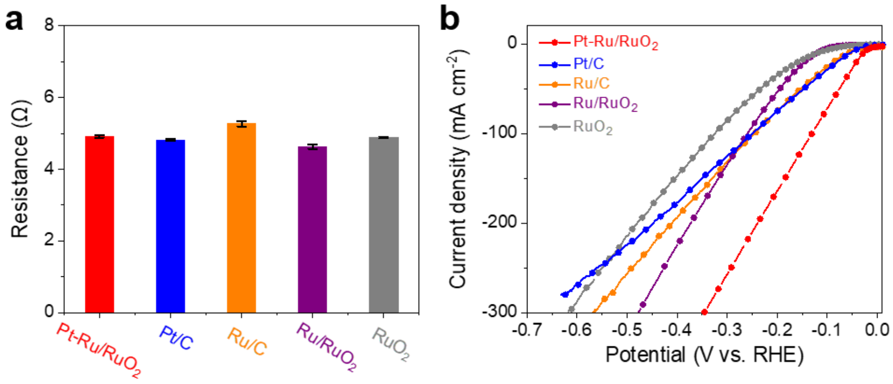

Supplementary Figure 13. Resistances and non-iR corrected LSVs of electrocatalysts. (a) The detected resistance for all studied electrocatalysts in 1 M KOH solution. Note: error bars represent the standard deviation of three independent measurements. (b) The non-iR corrected LSVs for all electrocatalysts.

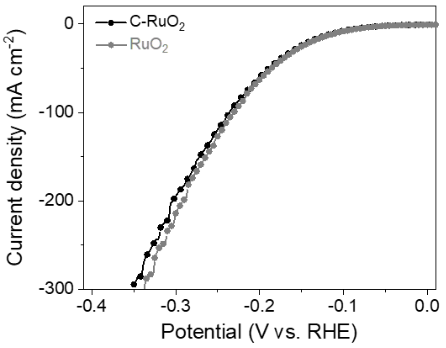

Supplementary Figure 14. LSVs of C-RuO_2 and synthesized RuO_2 in 1 M KOH solution.

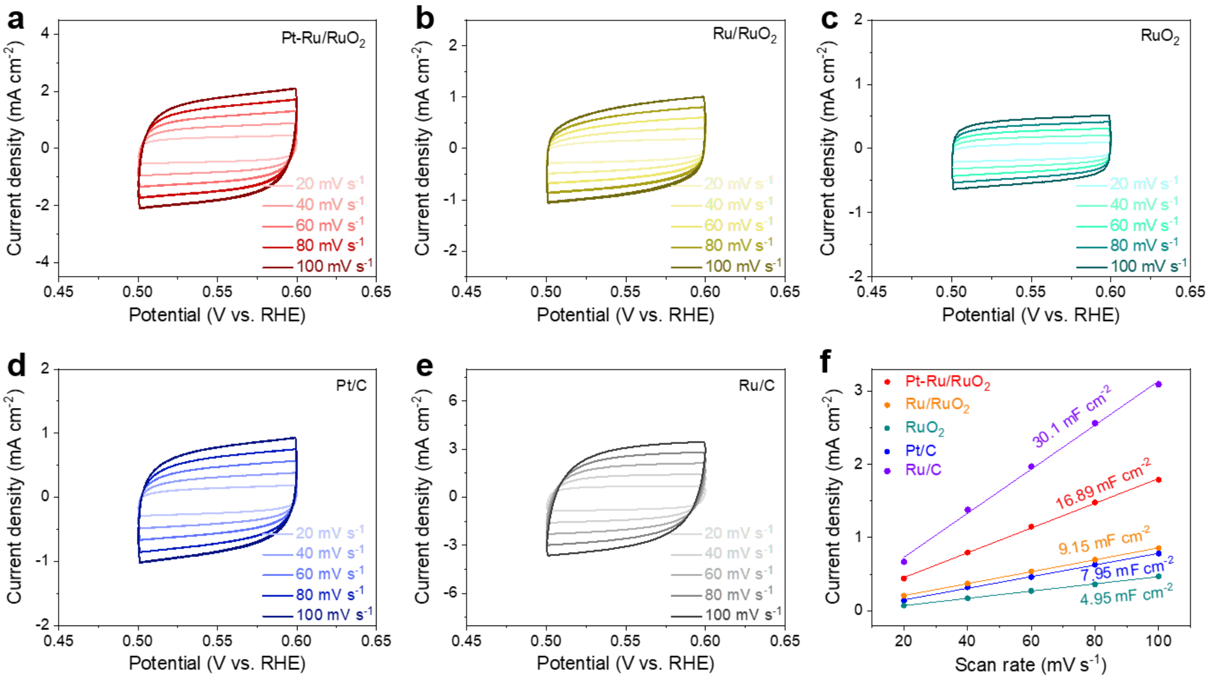

Supplementary Figure 15. **ECSA measurements of electrocatalysts.** Electrochemical cyclic voltammetry scans recorded for (a) Pt-Ru/RuO_2, (b) Ru/RuO_2, (c) RuO_2, (d) Pt/C and (e) Ru/C. Scan rates are 20, 40, 60, 80 and 100 mV s^{-1}. (f) Linear fitting of the capacitive currents versus cyclic voltammetry scans for these catalysts.

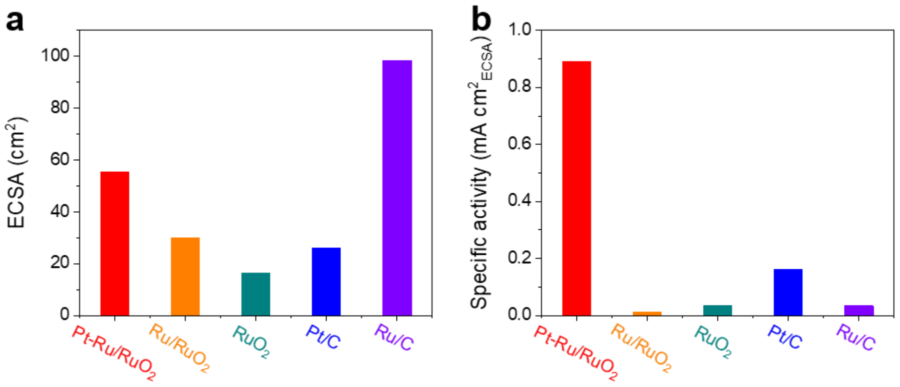

Supplementary Figure 16. **ECSA and specific activities of electrocatalysts.** The calculated (a) ECSA values and (b) specific activities at the overpotential of 63 mV on Pt-Ru/RuO_2, Ru/RuO_2, RuO_2, Pt/C and Ru/C.

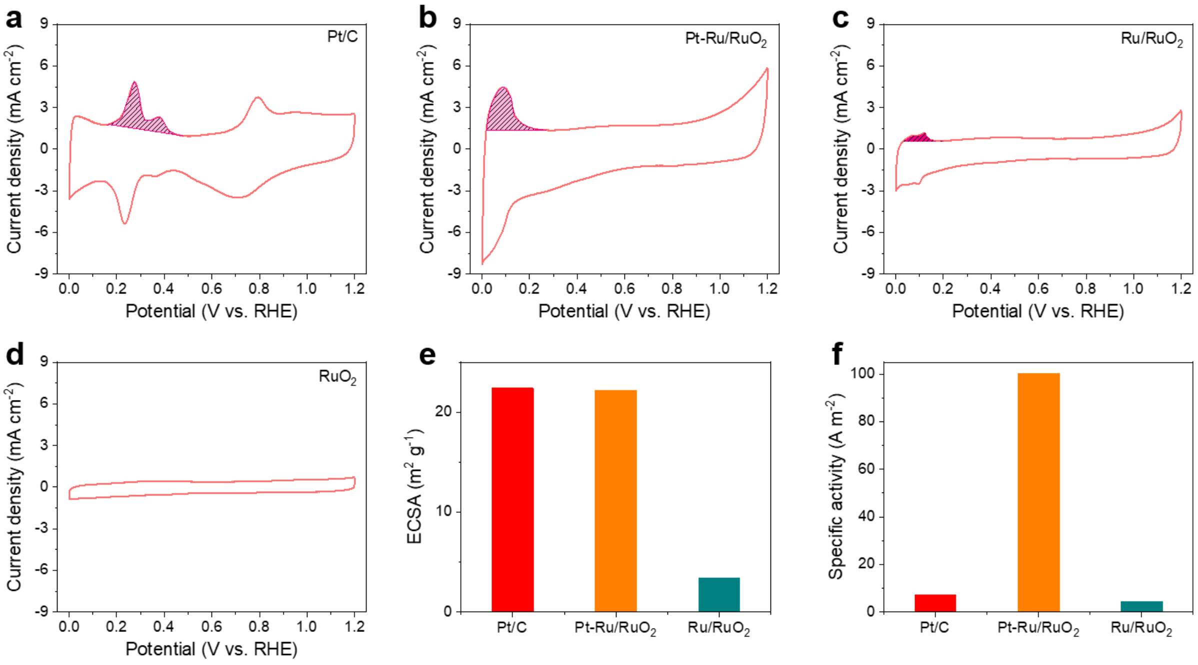

Supplementary Figure 17. **ECSA measurements and specific activities based on H_{upd} method.** The CV curves of (a) Pt/C, (b) Pt-Ru/RuO_2, (c) Ru/RuO_2 and (d) RuO_2, the scan rate was controlled as 50 mV s^{-1}. (e) The corresponding ECSA values and (f) specific activities of Pt/C, Pt-Ru/RuO_2 and Ru/RuO_2 derived from the H_{upd} method.

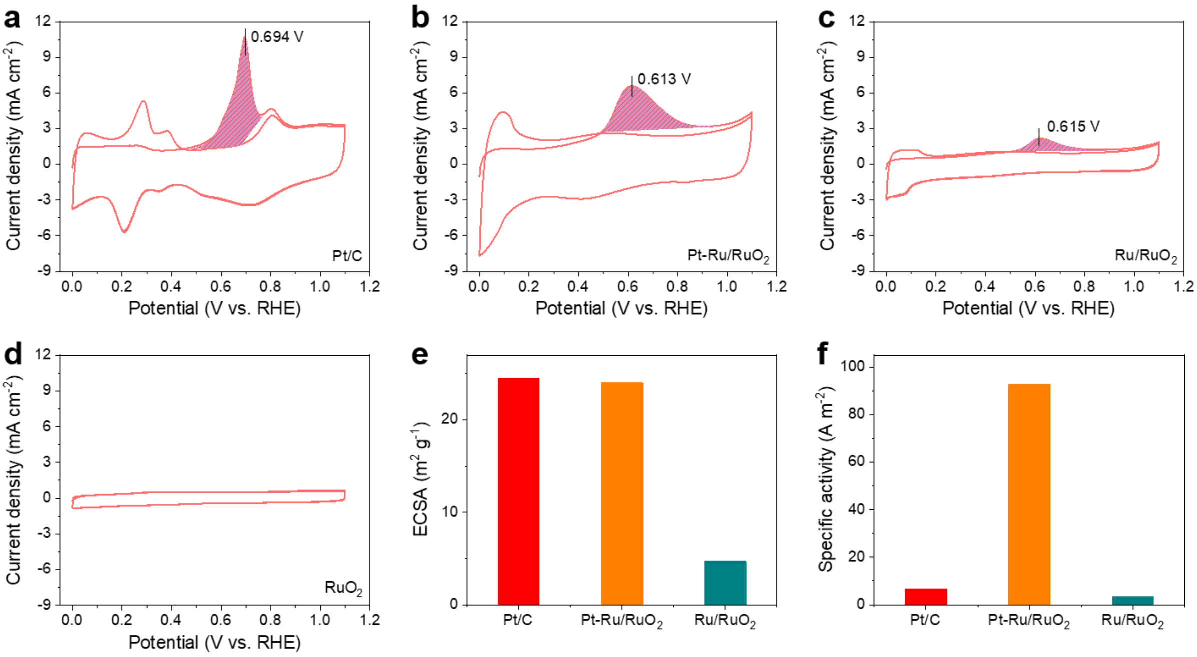

Supplementary Figure 18. **ECSA measurements and specific activities based on CO stripping method.** The CO stripping curves of (a) Pt/C, (b) Pt-Ru/RuO_2, (c) Ru/RuO_2 and (d) RuO_2, the scan rate was controlled as 50 mV s^{-1}. (e) The corresponding ECSA values and (f) specific activities of Pt/C, Pt-Ru/RuO_2 and Ru/RuO_2 derived from the CO stripping method.

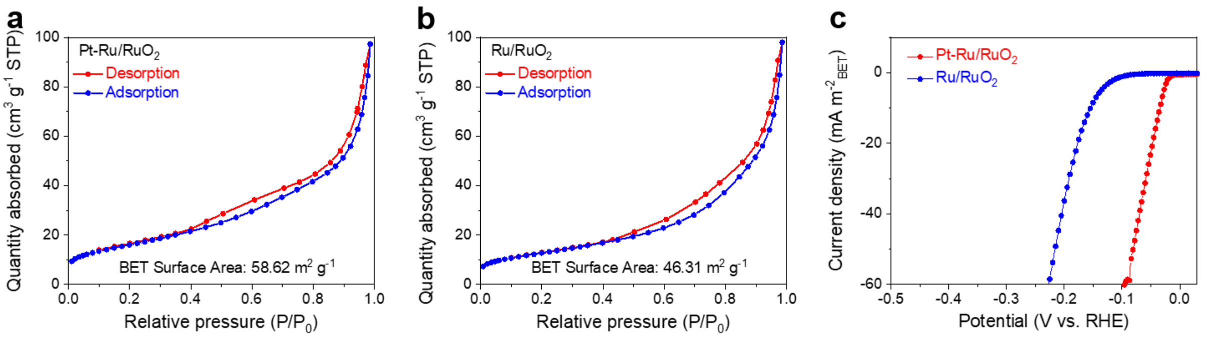

Supplementary Figure 19. **BET measurements and normalized LSVs.** BET surface areas of (a) Pt-Ru/RuO_2 and (b) Ru/RuO_2. (c) Corresponding LSV curves normalized to the BET surface areas.

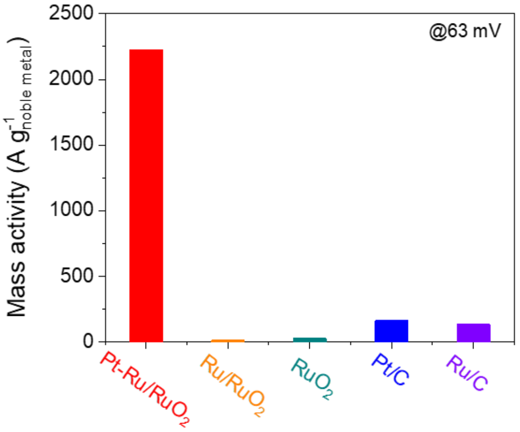

Supplementary Figure 20. **Mass activities of electrocatalysts.** Mass activities of Pt-Ru/RuO_2, Ru/RuO_2, RuO_2, Pt/C and Ru/C by normalizing the currents at the overpotential of 63 mV to the masses of noble metals.

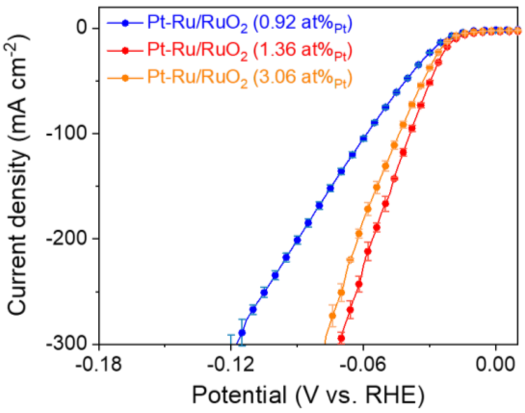

Supplementary Figure 21. **LSVs of Pt-Ru/RuO_2 with various Pt doping amounts.** The polarization curves of Pt-Ru/RuO_2 with different Pt doping amount (0.92 at%, 1.36 at% and 3.06 at%) in 1 M KOH solution. Note: error bars represent the standard deviation of three independent measurements.

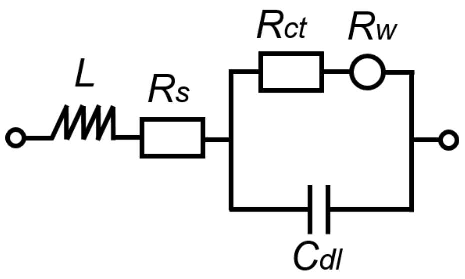

Supplementary Figure 22. **The equivalent circuit model applied in the EIS tests.**

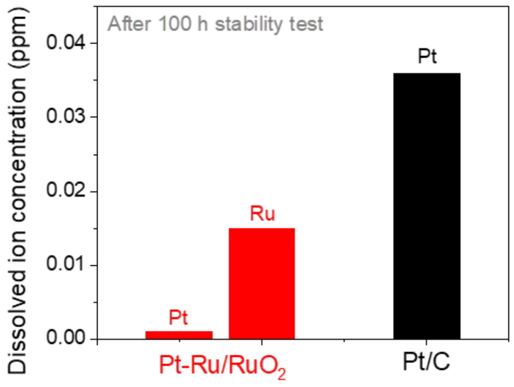

Supplementary Figure 23. **Ion leaching tests.** The dissolved Pt and Ru ion concentrations of Pt-Ru/RuO_2 and Pt/C after 100 h stability test at 10 mA cm^{-2} in the 1 M KOH electrolyte.

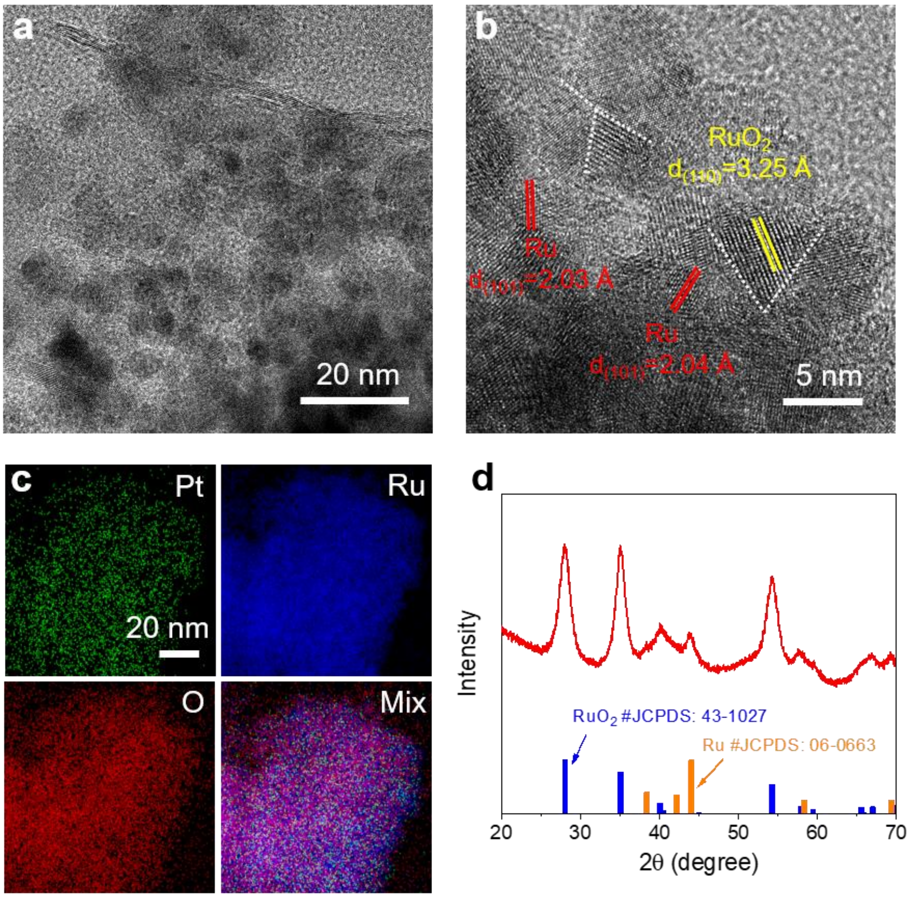

Supplementary Figure 24. **Characterizations of Pt-Ru/RuO_2 after stability test.** The (a) TEM, (b) HR-TEM images, (c) elemental mappings and (d) XRD pattern of Pt-Ru/RuO_2 after 100 h stability test at 10 mA cm^{-2}.

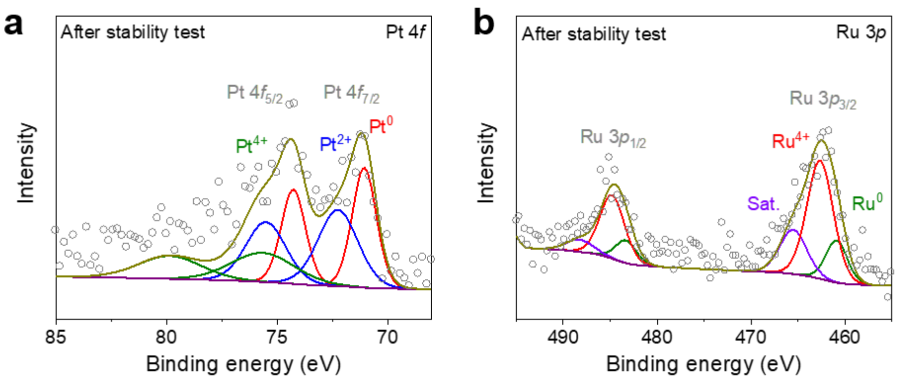

Supplementary Figure 25. **XPS spectra of Pt-Ru/RuO_2 after stability test.** The (a) Pt 4*f* and (b) Ru 3*p* XPS spectra of Pt-Ru/RuO_2 after 100 h stability test at 10 mA cm^{-2}.

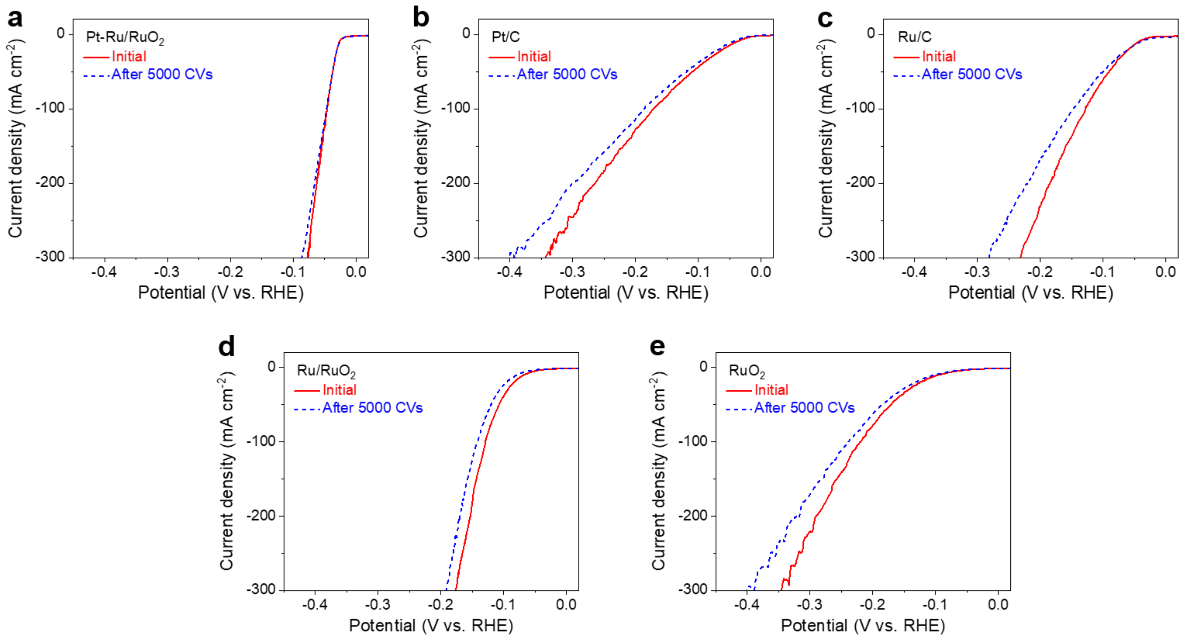

Supplementary Figure 26. LSVs before and after stability test. Polarization curves of (a) Pt-Ru/RuO_2, (b) Pt/C, (c) Ru/C, (d) Ru/RuO_2 and (e) RuO_2 before and after 5000 CV cycles.

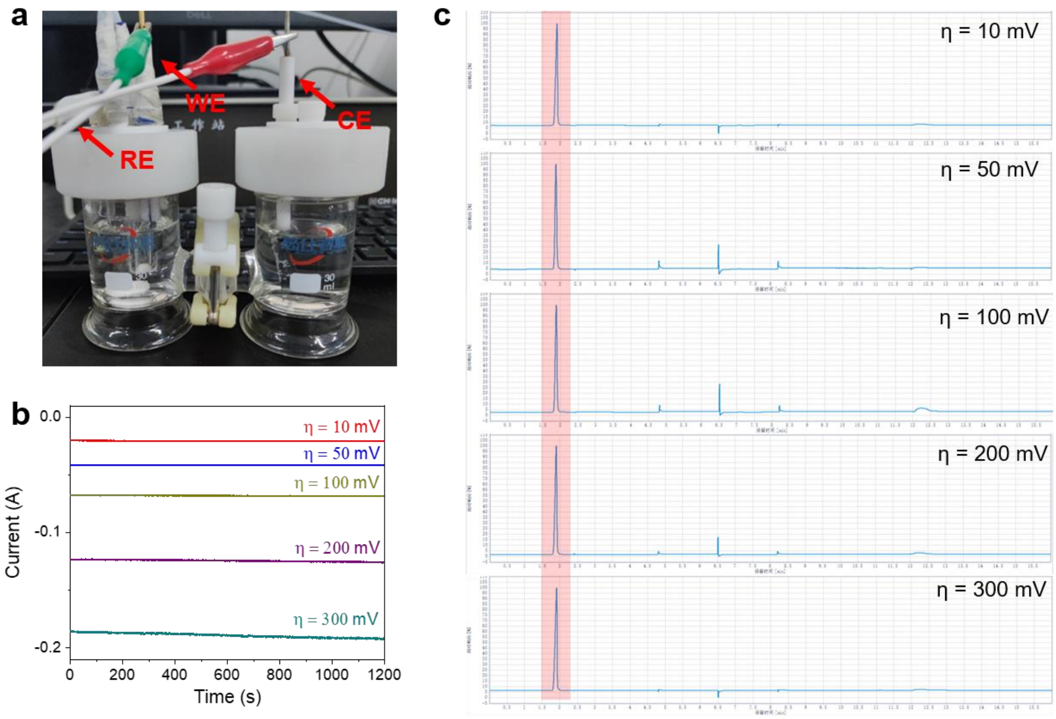

Supplementary Figure 27. GC measurement of Pt-Ru/RuO_2. (a) The electrochemical cell used for conducting the HER on Pt-Ru/RuO_2, which was connected to GC and Ar was used as the carrier gas to detect the amount of generated H_2. (b) Corresponding i-t curves of Pt-Ru/RuO_2 under various overpotentials for 1200 s. (c) The GC signals of generated H_2 amount detected under various overpotentials on Pt-Ru/RuO_2.

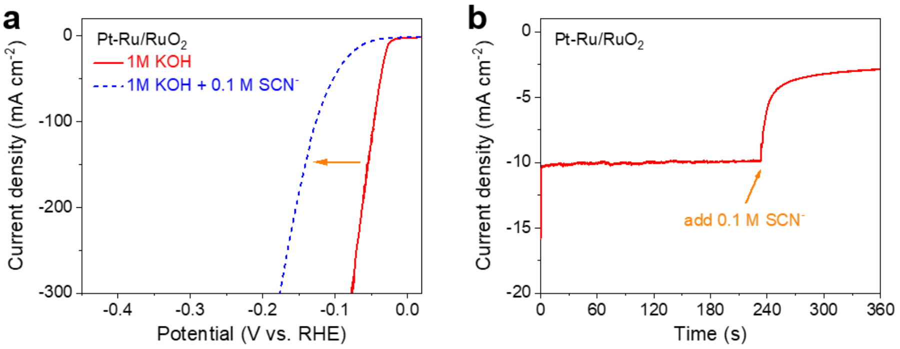

Supplementary Figure 28. **Poisoning tests of Pt-Ru/RuO_2.** (a) Polarization curves and (b) i-t curve of Pt-Ru/RuO_2 before and after adding the poisoning SCN^-.

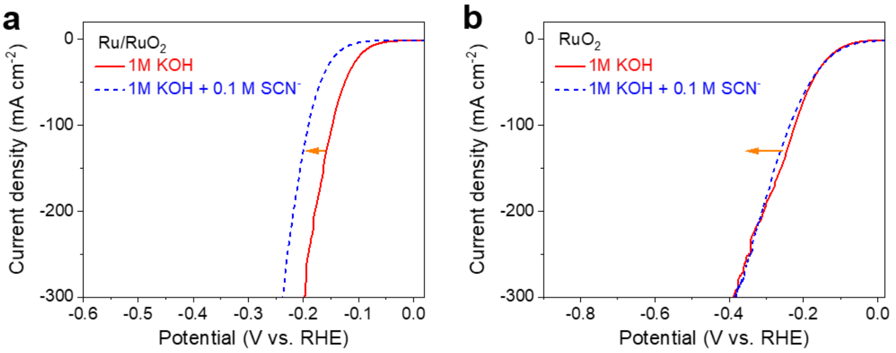

Supplementary Figure 29. **Poisoning tests of Ru/RuO_2 and RuO_2.** Polarization curves of (a) Ru/RuO_2 and (b)RuO_2 before and after adding the poisoning SCN^-.

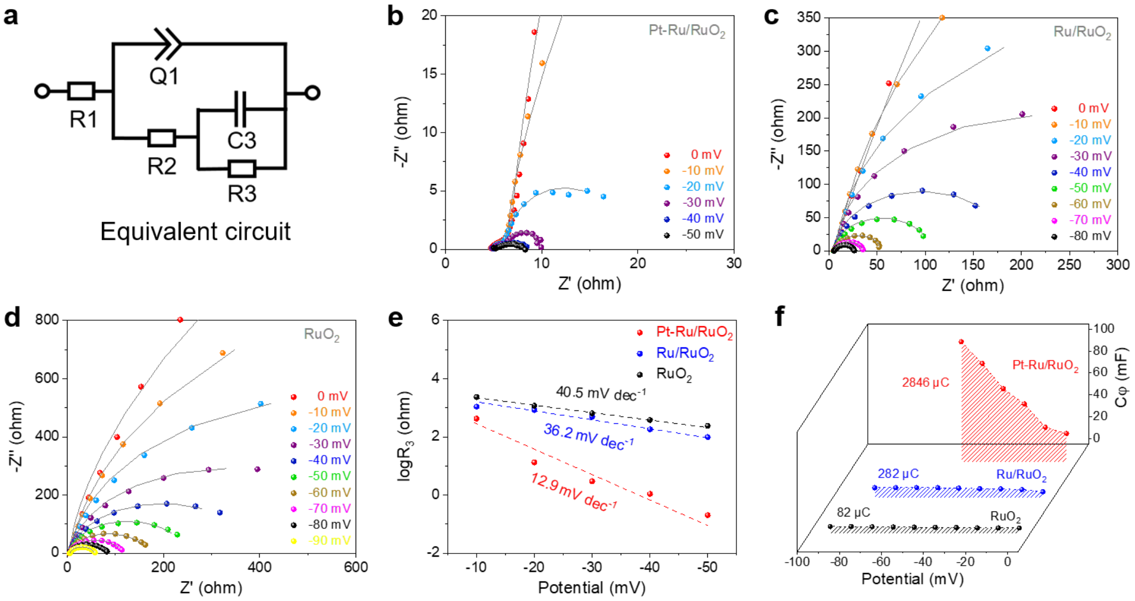

Supplementary Figure 30. **Operando EIS tests of Pt-Ru/RuO_2 and references.** (a) The adopted double-parallel equivalent circuit model. Corresponding Nyquist plots of (b) Pt-Ru/RuO_2, (c) Ru/RuO_2 and (d) RuO_2 at various applied potentials. (e) EIS-derived Tafel plots and (f) hydrogen adsorption pseudocapacitance as a function of potential for Pt-Ru/RuO_2, Ru/RuO_2 and RuO_2 obtained from fitting results.

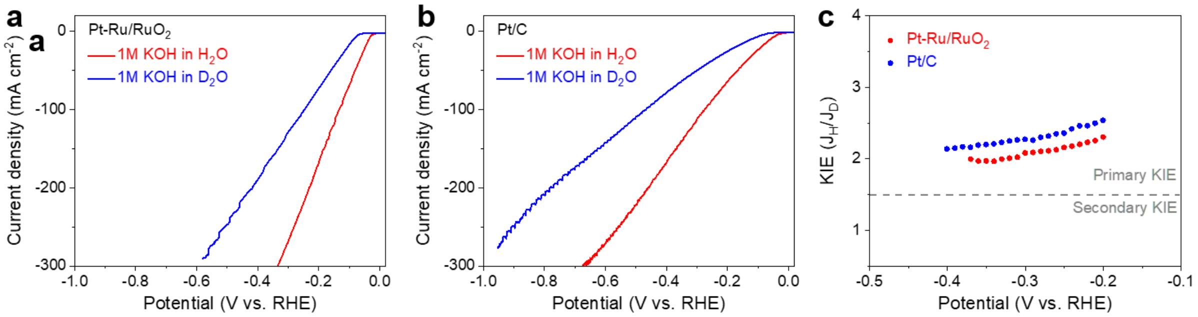

Supplementary Figure 31. **KIE measurements of Pt-Ru/RuO$_2$ and Pt/C.** The polarization curves of (a) Pt-Ru/RuO$_2$ and (b) Pt/C recorded in H_2O and D_2O electrolytes, respectively. (c) Corresponding KIE values of Pt-Ru/RuO$_2$ and Pt/C.

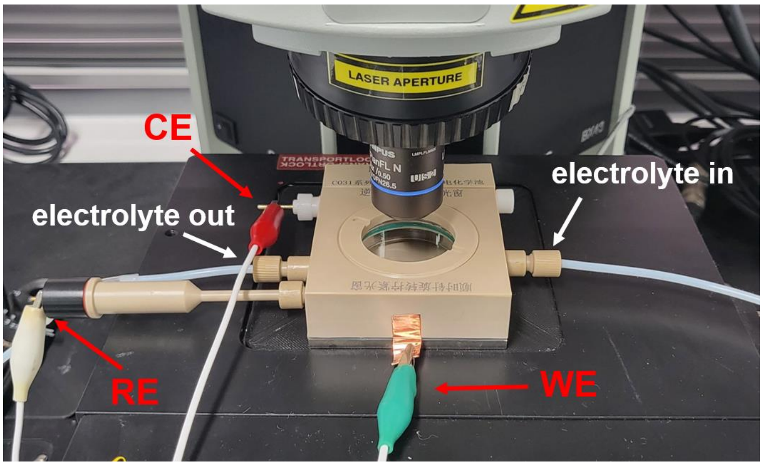

Supplementary Figure 32. The photo of the electrochemical cell for operando Raman tests.

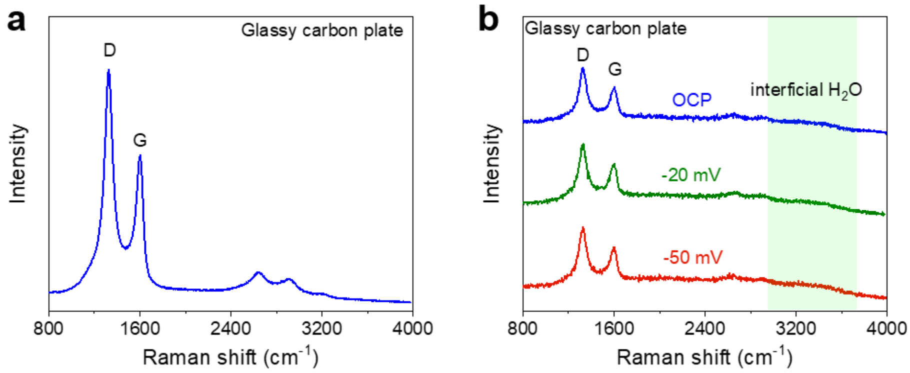

Supplementary Figure 33. **Raman spectra of glassy carbon plate.** (a) The ex-situ Raman spectrum of the glassy carbon plate without catalyst and electrolyte. (b) The operando Raman spectra of the glassy carbon plate in the electrolytes under various applied potentials.

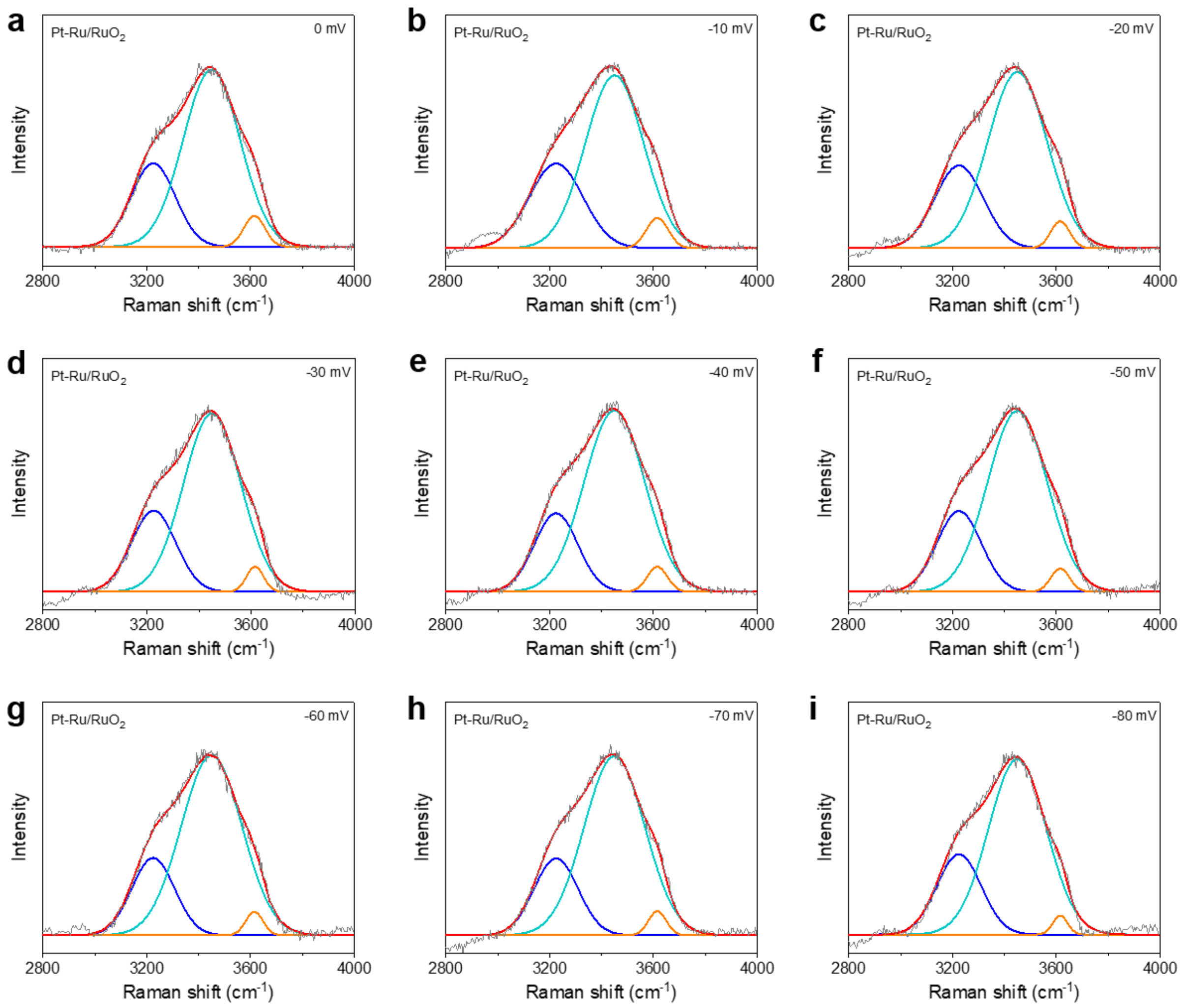

Supplementary Figure 34. **The fitting results of the interfacial water peaks in the Raman spectra**. The fitting spectra of the interfacial water peaks in the Raman spectra of Pt-Ru/RuO_2 at (a) 0 mV, (b) -10 mV, (c) -20 mV, (d) -30 mV, (e) -40 mV, (f) -50 mV, (g) -60 mV, (h) -70 mV, (i) -80 mV, respectively.

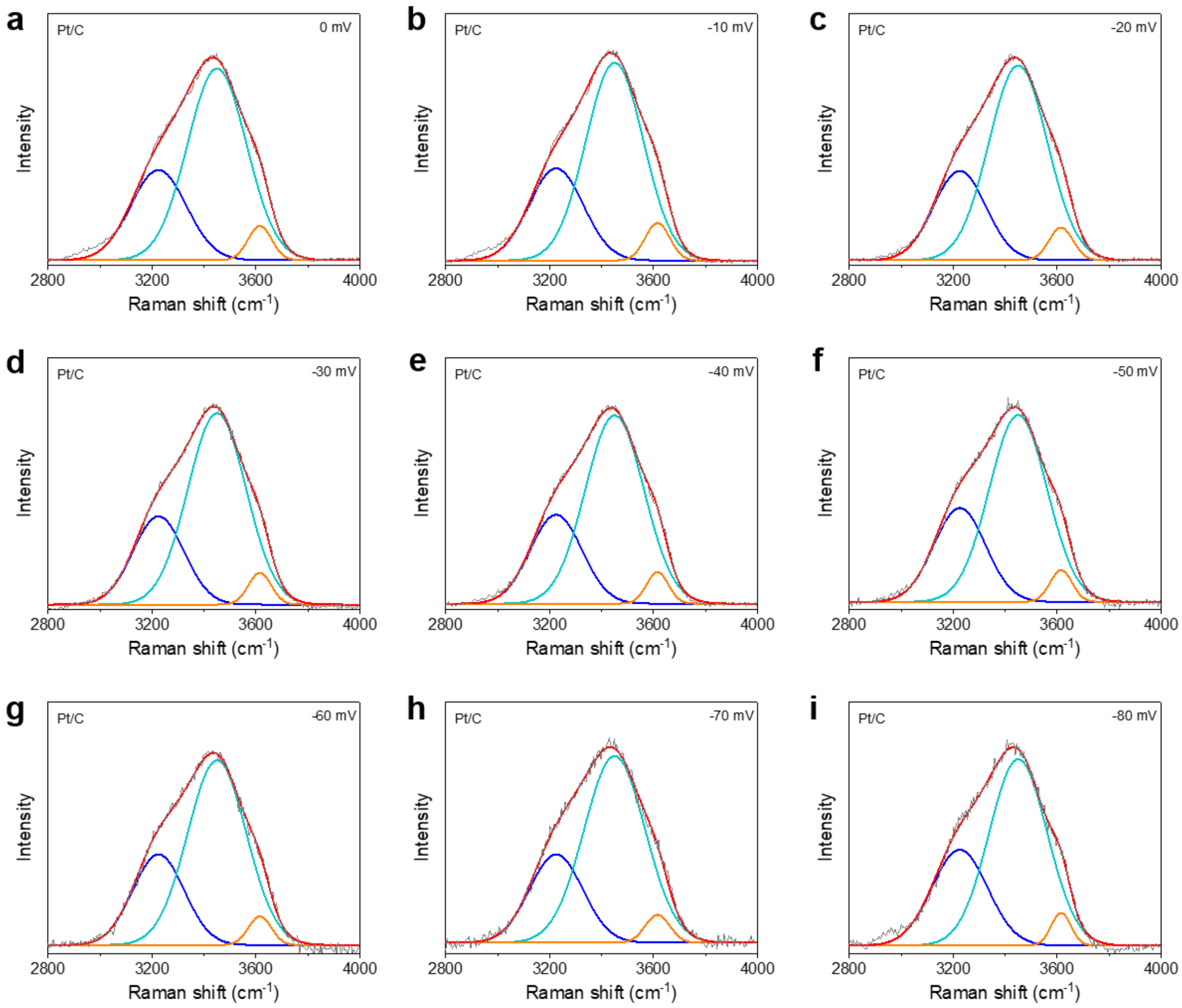

Supplementary Figure 35. **The fitting results of the interfacial water peaks in the Raman spectra**. The fitting spectra of the interfacial water peaks in the Raman spectra of Pt/C at (a) 0 mV, (b) -10 mV, (c) -20 mV, (d) -30 mV, (e) -40 mV, (f) -50 mV, (g) -60 mV, (h) -70 mV, (i) -80 mV, respectively.

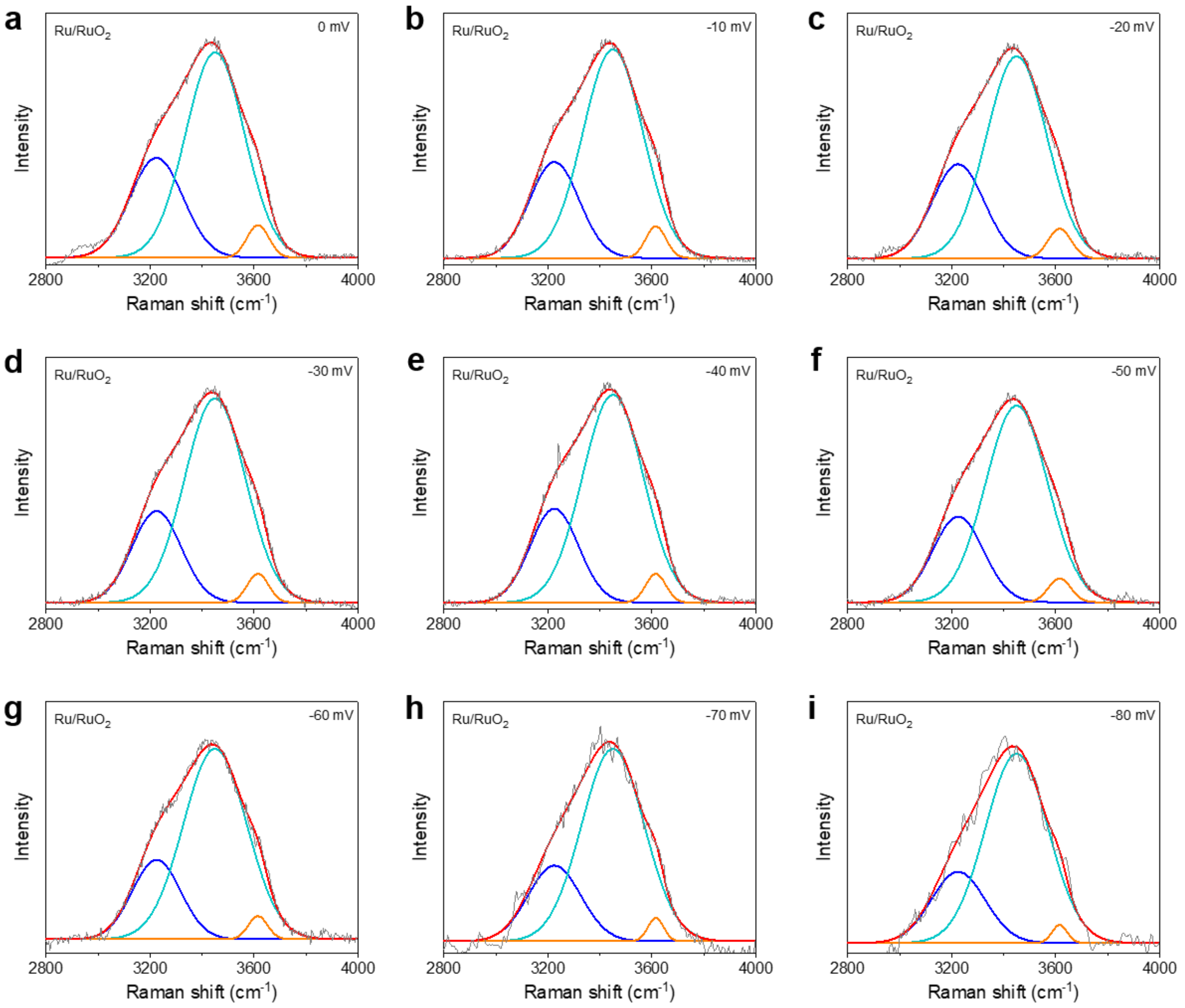

Supplementary Figure 36. **The fitting results of the interfacial water peaks in the Raman spectra**. The fitting spectra of the interfacial water peaks in the Raman spectra of Ru/RuO_2 at (a) 0 mV, (b) -10 mV, (c) -20 mV, (d) -30 mV, (e) -40 mV, (f) -50 mV, (g) -60 mV, (h) -70 mV, (i) -80 mV, respectively.

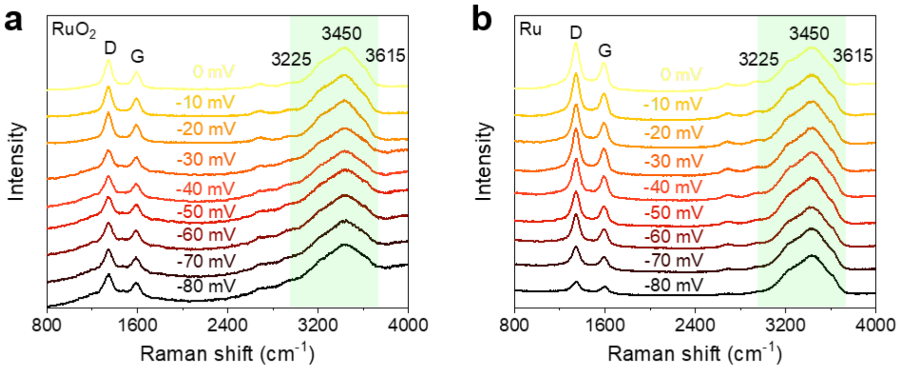

Supplementary Figure 37. **Operando Raman tests on electrocatalysts.** The operando Raman spectra of (a) RuO_2 and (b) Ru under applied potentials in 1 M KOH solution.

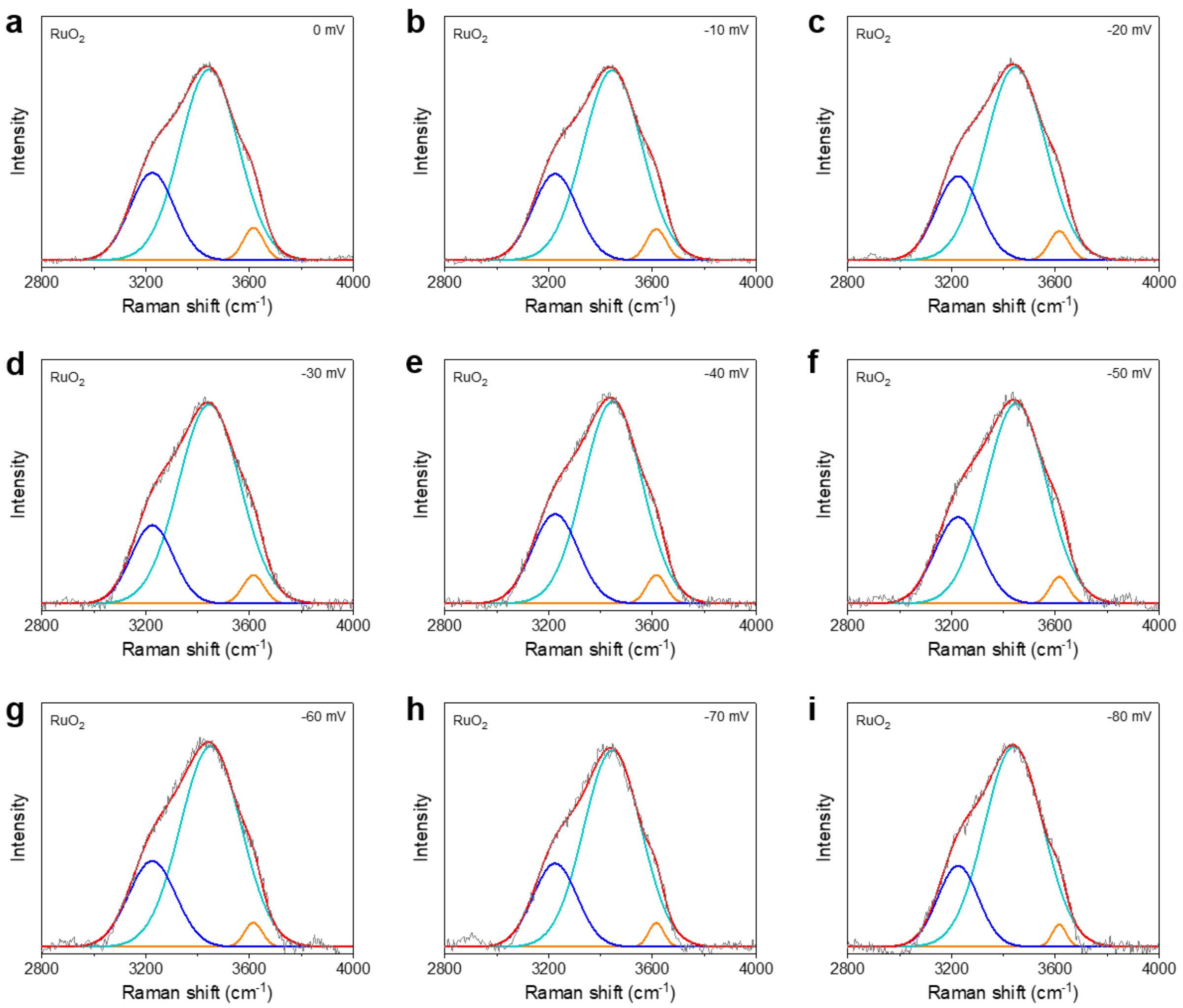

Supplementary Figure 38. **The fitting results of the interfacial water peaks in the Raman spectra**. The fitting spectra of the interfacial water peaks in the Raman spectra of RuO_2 at (a) 0 mV, (b) -10 mV, (c) -20 mV, (d) -30 mV, (e) -40 mV, (f) -50 mV, (g) -60 mV, (h) -70 mV, (i) -80 mV, respectively.

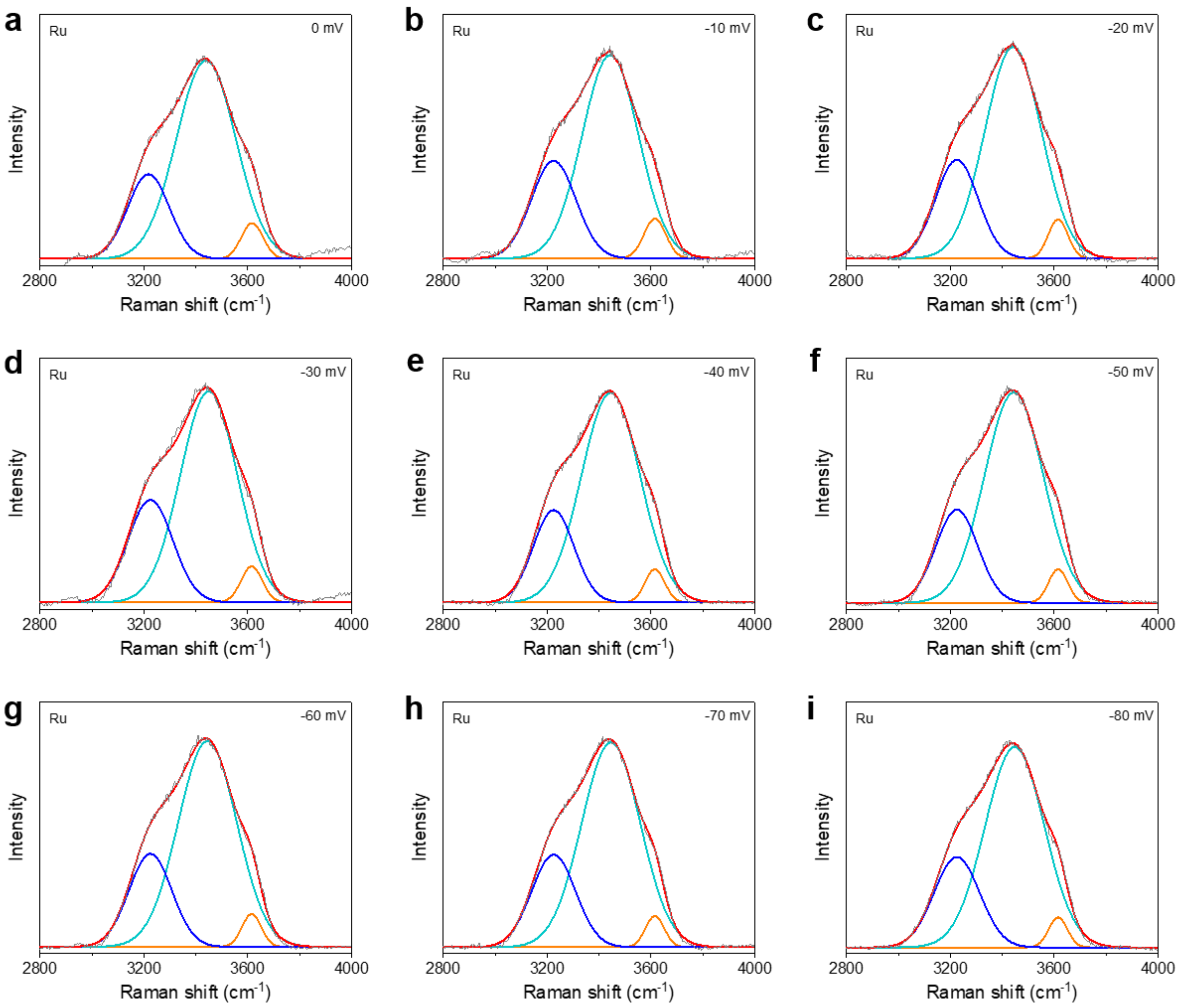

Supplementary Figure 39. **The fitting results of the interfacial water peaks in the Raman spectra**. The fitting spectra of the interfacial water peaks in the Raman spectra of Ru at (a) 0 mV, (b) -10 mV, (c) -20 mV, (d) -30 mV, (e) -40 mV, (f) -50 mV, (g) -60 mV, (h) -70 mV, (i) -80 mV, respectively.

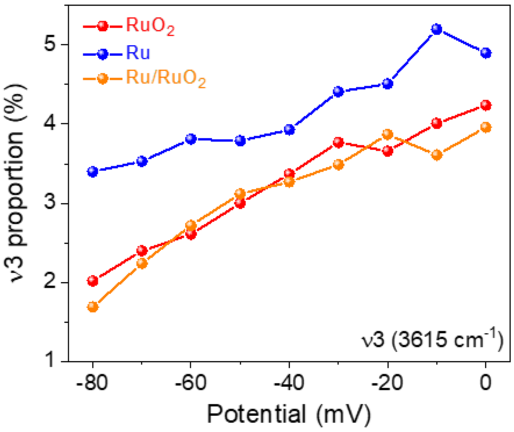

Supplementary Figure 40. **The evolution of υ3 peaks proportions.** The proportions of υ3 peaks at 3615 cm^{-1} during HER on RuO_2, Ru and Ru/RuO_2.

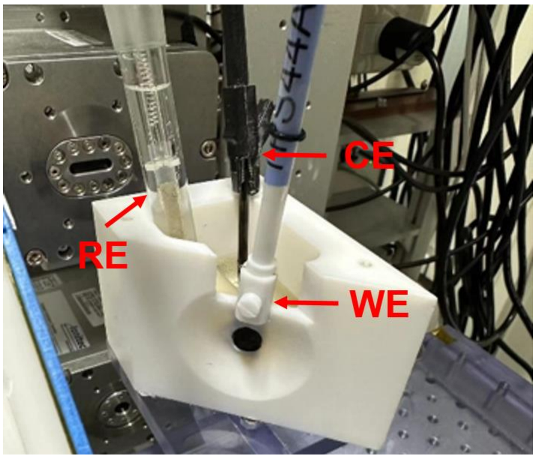

Supplementary Figure 41. The photo of the custom-built electrochemical cell for operando XAS test.

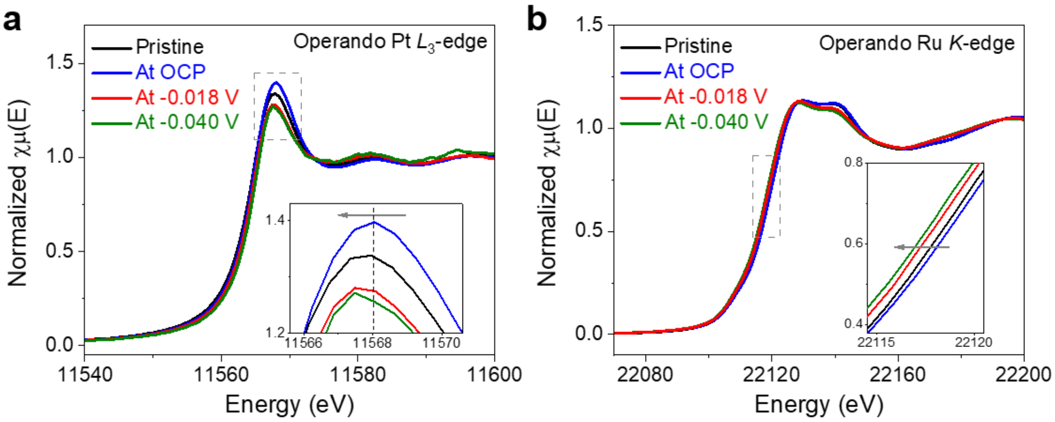

Supplementary Figure 42. Operando XANES tests on Pt-Ru/RuO$_2$. The (a) Pt L_3-edge and (b) Ru K-edge XANES spectra of Pt-Ru/RuO$_2$ measured in 1 M KOH solution under pristine, OCP and HER operating conditions of -0.018 V and -0.040 V.

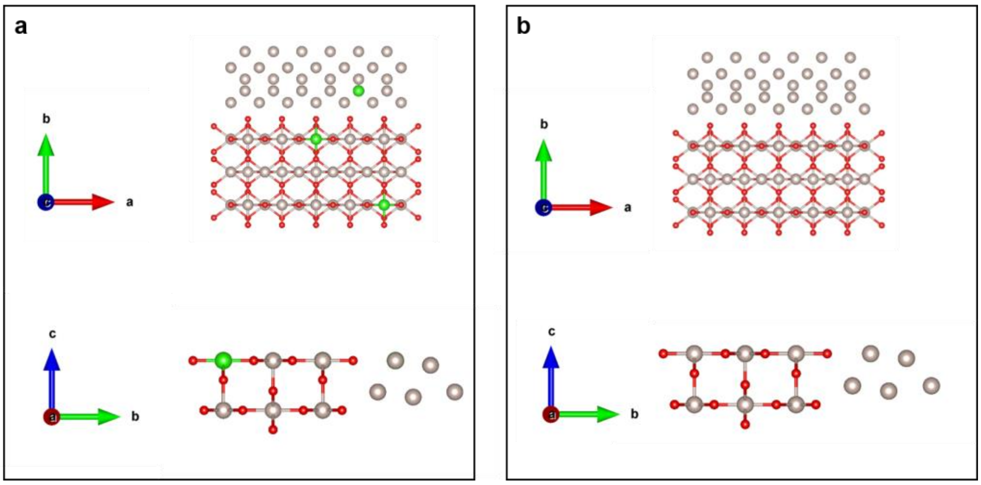

Supplementary Figure 43. DFT models of Pt-Ru/RuO$_2$ and Ru/RuO$_2$. The established atomic models of (a) Pt-Ru/RuO$_2$ and (b) Ru/RuO$_2$ for DFT calculations. The Pt, Ru and O atoms were shown in green, grey and red, respectively.

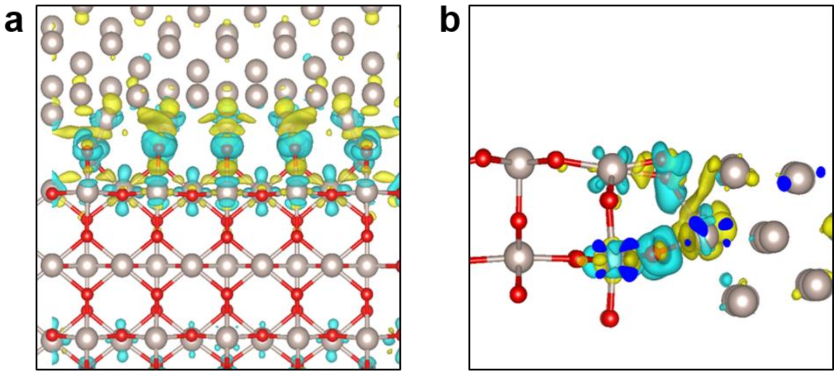

Supplementary Figure 44. **Charge density difference of Ru/RuO_2.** The (a) top view and (b) side view of charge density difference for Ru/RuO_2 surface model. Blue and yellow regions represent the electron-accumulated region and the electron-depleted region, respectively.

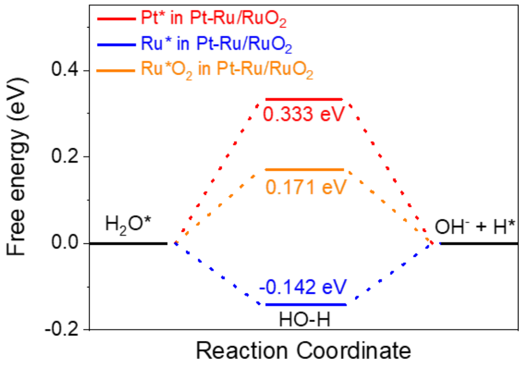

Supplementary Figure 45. **The H_2O^* dissociation free energy diagram.** The H_2O^* dissociation free energy for Pt, Ru and RuO_2 sites in Pt-Ru/RuO_2.

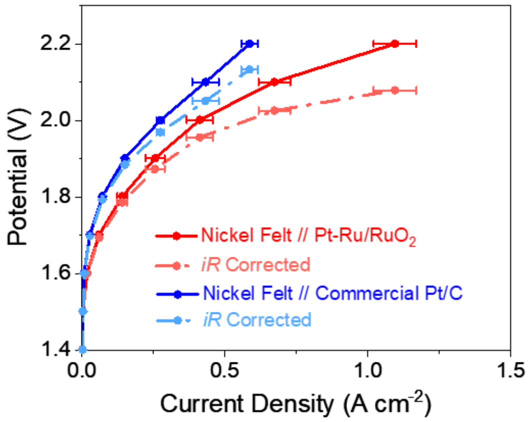

Supplementary Figure 46. **AEMWE performances.** The AEMWE polarization curves with and without iR correction on Ni felt // Pt-Ru/RuO_2 and Ni felt // commercial Pt/C. Note: error bars represent the standard deviation of three independent measurements.

Supplementary Table 1. Results of XRD refinements for Pt-Ru/RuO_2 and Ru/RuO_2.

Compounds	Phases	*a* (Å)	*b* (Å)	*c* (Å)	*V* (Å^3)	Fraction
Pt-Ru/RuO_2	RuO_2	4.532	4.532	3.122	64.143	74.82%
	Ru	2.715	2.715	4.396	28.060	25.18%
Ru/RuO_2	RuO_2	4.528	4.528	3.114	63.835	84.97%
	Ru	2.694	2.694	4.407	27.693	15.03%

Supplementary Table 2. Fit goodness and R-factor of XRD refinements for Pt-Ru/RuO_2 and Ru/RuO_2.

Compounds	Phases	χ^2	R_F	R_B	R_P	R_{WP}
Pt-Ru/RuO_2	RuO_2	2.40	0.632%	0.783%	2.31%	3.17%
	Ru	2.40	1.230%	1.230%	2.31%	3.17%
Ru/RuO_2	RuO_2	2.67	0.077%	0.101%	1.82%	2.45%
	Ru	2.67	0.634%	1.210%	1.82%	2.45%

Supplementary Table 3. EXAFS fitting parameters of Pt-Ru/RuO_2, Pt foil and PtO_2.

Compounds	Path	N	S02	σ^2 / Å^2	R / Å	ΔE0	R-factor
Pt-Ru/RuO_2	Pt-O1	3.9	0.633	0.0061±0.0085	2.049±0.005	16.3	0.01
	Pt-Ru1	1.4	0.671	0.0048±0.005	3.151±0.1845		
	Pt-Ru2	5.5	0.671	0.0188±0.1364	3.654±0.2025		
Pt foil	Pt-Pt	12	0.779	0.0042±0.0003	2.756±0.002	6.9	0.002
PtO_2	Pt-O	6	0.975	0.0035±0.0021	2.013±0.015	9.0	0.012

Supplementary Table 4. Comparisons of the Tafel slopes and overpotentials at the current density of 10 mA cm^{-2} of reported Pt, Ru-based catalysts for HER in 1 M KOH solution.

Catalysts	Overpotential (mV)	Tafel slope (mV dec^{-1})	Ref.
CoPt-Pt_{SA}	31	43.65	1
D-NiO-Pt	20	31.1	2
Ni_5P_4-Ru	54	52	3
Pt_{SA}-Mn_3O_4	24	54	4
Pt@DG	37	53	5
2D-Pt ND/LDH	25	32.2	6
Ru/np-MoS_2	30	31	7
Pt_{SA}-NiO/Ni	26	27.07	8
Vo-Ru/HfO_2-OP	39	29	9
Commercial Pt/C	45	50.7	**This work**
Commercial Ru/C	49	64.0	
**Pt-Ru/RuO_2**	**18**	**18.5**	

Supplementary Table 5. Fitting parameters of EIS for Pt-Ru/RuO_2, Pt/C, Ru/C, Ru/RuO_2 and RuO_2.

Samples	R_s (Ω)	R_{ct} (Ω)	C_{dl} (F s^{n-1})	s (Ω $s^{-1/2}$)
Pt-Ru/RuO_2	3.8	0.42	0.0045	0.05
Pt/C	4.2	10.4	0.0005	0.02
Ru/C	5.1	21.7	0.001	0.0001
Ru/RuO_2	4.5	121.2	0.001	0.0001
RuO_2	4.3	196.8	0.0015	0.0001

Supplementary Table 6. The fitting parameters of operando EIS for Pt-Ru/RuO_2, Ru/RuO_2 and RuO_2.

Samples	Potential (mV)	R_1 (Ω)	R_2 (Ω)	R_3 (Ω)	C_3 (mF)
Pt-Ru/RuO_2	0	4.7	3.2	1520	21.0
	-10	4.8	2.6	424.1	26.6
	-20	4.8	2.0	13.4	48.0
	-30	4.8	2.2	3.0	62.0
	-40	4.9	2.2	1.3	85.0
	-50	5.0	3.0	0.2	105.4
Ru/RuO_2	0	4.8	10.21	2897	0.49
	-10	4.9	7.2	1120	2.56
	-20	5.0	4.4	834.2	3.20
	-30	4.9	2.06	496.2	3.44
	-40	4.9	1.45	180.5	3.89
	-50	5.0	0.75	98.8	4.29
	-60	4.9	1.36	48.4	4.27
	-70	5.0	0.92	29.4	4.28
	-80	5.0	0.7	20.5	4.22
RuO_2	0	5.0	3.23	9437	0.23
	-10	5.1	3.72	2369	0.35
	-20	5.1	2.3	1184	0.41
	-30	5.1	2.10	650.0	0.69
	-40	5.1	1.40	388.9	0.87
	-50	5.2	1.41	239.9	0.94
	-60	4.9	1.54	169.7	1.14
	-70	5.0	1.28	110.6	1.36
	-80	5.1	1.22	76.3	1.53
	-90	5.2	1.28	50.3	1.65

Supplementary Table 7. Fitted ʋ3 fractions in operando Raman for Pt-Ru/RuO_2, Pt/C, Ru/RuO_2, RuO_2 and Ru.

Potentials (mV)	0	-10	-20	-30	-40	-50	-60	-70	-80
ʋ3 fraction (Pt-Ru/RuO_2)	4.6%	4.3%	3.4%	3.0%	3.3%	2.9%	2.8%	2.9%	2.0%
ʋ3 fraction (Pt/C)	4.7%	5.3%	4.7%	4.5%	4.2%	4.3%	4.1%	4.0%	3.9%
ʋ3 fraction (Ru/RuO_2)	4.0%	3.6%	3.9%	3.5%	3.3%	3.1%	2.7%	2.2%	1.7%
ʋ3 fraction (RuO_2)	4.2%	4.0%	3.7%	3.8%	3.4%	3.0%	2.6%	2.4%	2.0%
ʋ3 fraction (Ru)	4.9%	5.2%	4.5%	4.4%	3.9%	3.7%	3.8%	3.5%	3.4%

Supplementary References

1. Yang, W. et al. Tuning the cobalt-platinum alloy regulating single-atom platinum for highly efficient hydrogen evolution reaction. *Adv. Funct. Mater.* **32**, 2205920 (2022).
2. Yan, Y. et al. Atomic-level platinum filling into Ni-vacancies of dual-deficient NiO for boosting electrocatalytic hydrogen evolution. *Adv. Energy Mater.* **12**, 2200434 (2022).
3. He, Q. et al. Achieving efficient alkaline hydrogen evolution reaction over a Ni_5P_4 catalyst incorporating single-atomic Ru sites. *Adv. Mater.* **32**, 1906972 (2020).
4. Wei, J. et al. In situ precise anchoring of Pt single atoms in spinel Mn_3O_4 for a highly efficient hydrogen evolution reaction. *Energy Environ. Sci.* **15**, 4592 (2022)
5. Yang, Q. et al. Single carbon vacancy traps atomic platinum for hydrogen evolution catalysis. *J. Am. Chem. Soc.* **144**, 2171-2178 (2022).
6. Hong, Y.-R. et al. Crystal facet-manipulated 2D Pt nanodendrites to achieve an intimate heterointerface for hydrogen evolution reactions. *J. Am. Chem. Soc.* **144**, 9033-9043 (2022).
7. Jiang, K. et al. Rational strain engineering of single-atom ruthenium on nanoporous MoS_2 for highly efficient hydrogen evolution. *Nat. Commun.* **12**, 1687 (2021).
8. Zhou, K. L. et al. Platinum single-atom catalyst coupled with transition metal/metal oxide heterostructure for accelerating alkaline hydrogen evolution reaction. *Nat. Commun.* **12**, 3783 (2021).
9. Li, G. et al. The synergistic effect of Hf-O-Ru bonds and oxygen vacancies in Ru/HfO_2 for enhanced hydrogen evolution. *Nat. Commun.* **13**, 1270 (2022).
